# Younger age is associated with greater pain expression among patients with knee or hip osteoarthritis scheduled for a joint arthroplasty

**DOI:** 10.1186/s12891-019-2740-8

**Published:** 2019-08-07

**Authors:** Josefina Skogö Nyvang, Josefine E. Naili, Maura D. Iversen, Eva W. Broström, Margareta Hedström

**Affiliations:** 1 Department of Clinical Science, Intervention and Technology, Division of Orthopaedics and Biotechnology, Karolinska Institutet, Karolinska University Hospital, K54, 141 86 Stockholm, Sweden; 2 Capio Geriatrik Nacka, Lasarettsvägen 4, 131 83 Nacka, Sweden; 3 Department of Women’s and Children’s Health, Karolinska Intstitutet, Q2:07, Karolinska University Hospital, 171 76 Stockholm, Sweden; 4 Highly Specialized Paediatric Orthopaedic and Medicine, Karolinska University Hospital, 171 76 Stockholm, Sweden; 5 Department of Physical Therapy, Movement & Rehabilitation Sciences, Bouve College of Health Sciences, Northeastern University, 360 Huntington Avenue, Boston, MA 02115 USA; 6 Division of Rheumatology, Immunology & Allergy, Brigham & Women’s Hospital, Harvard Medical School, Boston, MA 02115 USA; 7 Reconstructive Orthopaedics, Karolinska University Hospital Huddinge, 141 86 Stockholm, Sweden

**Keywords:** Knee osteoarthritis, Hip osteoarthritis, Age differences, Sensory pain, Affective pain, Sex differences

## Abstract

**Background:**

This study describes how patients with knee or hip osteoarthritis (OA), scheduled for arthroplasty, characterize their pain qualitatively and quantitatively and investigates whether differences exist in pain expression between younger and older patients, and between men and women.

**Methods:**

One hundred eight patients scheduled for a joint arthroplasty completed the Knee Injury and Osteoarthritis Outcome Score (KOOS) or Hip Disability and Osteoarthritis Outcome Score (HOOS) and a health-related quality of life question. Pain was assessed using the visual analogue scale (VAS), KOOS/HOOS and the Pain-o-Meter (POM) consisting of 12 sensory and 11 affective words (POM-Words). Frequency of analgesics use was assessed and preoperative radiographs were graded. ANOVA was used to test differences in pain expression with age (< 65 vs. ≥65 years), sex, and affected joint as independent factors.

**Results:**

Patients < 65 years of age used more affective words (POM) and words with higher affective intensity (median scores 8 (3–39), 5.5 (2–27) respectively), than older patients, despite having less radiographically advanced OA. They also reported more symptoms (KOOS/HOOS) than older patients. However, pain ratings, as measured by VAS and KOOS/HOOS pain, did not differ between younger and older adults. Women reported more frequent analgesics use (45.7 and 26.5% respectively) and rated their pain higher than men (mean POM-VAS = 42 (SD 24) and 31 (SD 19); respectively). No differences existed between sexes for sensory or affective POM-Words, or radiographic grade of OA. With age and sex as independent factors, a significant difference between knee and hip OA remained for sensory POM-words intensity scores.

**Conclusions:**

Younger adults scheduled for arthroplasty expressed pain using more affective words and words with higher intensity and had less radiographically advanced OA than older adults. However, VAS and KOOS/HOOS pain subscales could not distinguish the difference in pain expression. Thus, the POM may be a valuable tool for assessment of pain.

## Background

Total joint arthroplasties (TJA) due to osteoarthritis (OA) are continuously increasing and the greatest rise is found among younger patients [1–3]; a fivefold increase between 1998 and 2007 in total knee arthroplasties (TKA) among patients under 55 years of age [4]. Higher body mass index (BMI) and population growth can only partly explain the increase in knee arthroplasties [3]. A rising incidence of OA and more severe OA, as well as a broadening of the indications for arthroplasty among younger patients have also been suggested as reasons for this increase [4]. However, younger age i.e. less than 65 years, is a well-known risk factor for prosthesis failure and complications [1, 2, 5]. One study showed that younger patients reported more pain before TKA and pain did not improve as significantly as it did among older patients postoperatively [6]. Researchers have suggested that younger patients (< 65 years) may be considered “too young” and hence wait longer and experience worse preoperative symptoms before being considered for surgery [7]. On the contrary, older age has been associated with worse self-reported pain and symptoms at 1- and 5 years after a knee arthroplasty compared to younger age [8]. If a discrepancy exists in pain expression between younger and older patients before and after TJA remains unclear and few studies have evaluated whether a difference in pain expression exists by age or gender. One hypothesis, based on our clinical experience, is that younger and older patients express pain differently and are affected differently by pain. Among mature adults, younger age (45–64 years) has been associated with more intense negative emotions in relation to chronic pain compared to older age (≥ 65 years of age) [9]. In this study, we investigate whether younger age is associated with different pain expression among adults with knee or hip OA scheduled for joint arthroplasty. To our knowledge, this question has not previously been studied. To investigate pain expression in-depth, complementary questionnaires including several aspects of pain may be useful, as pain is multifaceted and also the major indication for surgery.

Sex differences refer to biological differences whereas gender differences arise from sociocultural processes. Data indicate inequality exists by sex (biological) and by gender (sociocultural) in health care [10–12]. These differences persist across various health conditions. For example, in a large registry study, the authors found that women had longer waiting time for cataract surgery than men [11] and in a study of cardiac care, women with acute coronary syndrome tended to delay seeking care compared to men [12]. Within the orthopaedic literature, we find women with OA have worse joint-related pain, function and disability prior to knee or hip arthroplasty, and present with more advanced disease at the time of surgery [13–15]. In addition, some studies suggest that physicians are less likely to recommend a TJA to women, despite radiographic evidence of OA and their willingness to undergo surgery [16, 17]. The reason for this discrepancy in surgical recommendation is unclear but our clinical experience suggests it may be due to sex differences in pain expression. Thus, different expressions in pain and clinical presentation between women and men may also be present in OA.

### Purpose and hypothesis

This study aimed to investigate how patients with knee or hip OA, who were scheduled for joint arthroplasty, expressed their pain and whether differences existed in pain expression between younger and older adults (< 65 vs. ≥65 years of age), and by sex. We hypothesized that younger mature adults with knee or hip OA would express pain differently than older adults. Secondarily, we hypothesize that women would express pain differently than men.

## Methods

### Study design

This cross-sectional study included a convenience sample of 108 patients with knee or hip OA who were scheduled for primary knee (*n* = 58) or hip (*n* = 50) arthroplasty and met the American Society of Anaesthesiologists (ASA) classification for general health status [18] (grades 1–2, indicating overall healthy individuals), with a mean age of 66.3 years (SD 8.5).

Two thirds of the participants were women. Between the years of 2010 and 2012, patients were consented and recruited from two orthopaedic departments in Stockholm: OrthoCenter Stockholm Löwenströmska Hospital and Karolinska University Hospital. Patients were included if they: were scheduled for arthroplasty due to primary knee- or hip OA and were able to understand verbal and written information in Swedish. Patients were excluded if they had other diseases affecting lower limb function (e.g. diabetes and/or neurological disease). The surgery coordinator asked patients who met inclusion and exclusion criteria to participate. Patients who declined were not registered. Two experienced orthopaedic surgeons (MH, PG) independently assessed all preoperative radiographs using the modified Kellgren & Lawrence classification of OA. The modified Kellgren & Lawrence classification of OA expands the Kellgren & Lawrence radiographic classification [19], by incorporating joint space narrowing and subdividing grades 3 and 4 into 3a/b and 4a/b [20]. All patients were asked to describe the joint-related pain they experienced when walking from the waiting room, according to the Pain-o-Meter (POM), which includes a visual analogue scale (VAS) and sensory and affective words [21]. They were also instructed to complete the following patient reported outcome (PRO) questionnaires: Knee Injury and Osteoarthritis Outcome Score (KOOS) [22] or Hip Disability and Osteoarthritis Outcome Score (HOOS) [23] and the VAS dimension of EuroQol 5 Dimension (EQ-5D) [24] health-related quality of life (HRQoL) measure. The frequency of analgesics use was registered as “never”, “when needed” or “daily”. Outcomes were examined between younger and older adults (< 65 vs. ≥65 years) and by sex.

### Patient-reported outcomes (PROs)

#### Pain

The POM is a validated instrument used to describe pain in patients with different chronic diseases [21, 25, 26]. There are two components of the POM: POM-VAS and POM-Words. POM-VAS quantifies pain using a 0–100 vertical scale ranging from best to worst. POM-Words consists of 12 sensory- and 11 affective words to qualitatively describe pain (Table 1). Each word has an assigned intensity value, unknown to the patient, ranging from 1 to 5 where 1 is considered a lighter pain than 5. The values are added to form intensity scores: one score for sensory words and one for affective words. Patients were allowed to choose as many words from the sensory- and the affective groups as necessary to describe their pain. They were instructed to characterize their pain using the sensory words and further to select the affect words that expressed how the pain made them feel emotionally (Table 1).

**Table 1:** Sensory and affective Pain-O-Meter Words [27]

Sensory words [intensity value]	Affective words [intensity value]
A Cutting [5]	M Irritating [2]
B Grinding [2]	N Frightening [4]
C Pricking [1]	O Troublesome [3]
D Squeezing [2]	P Suffocating [5]
E Cramping [4]	Q Killing [5]
F Tearing [5]	R Unbearable [4]
G Aching [3]	S Terrible [5]
H Smarting [2]	T Tiring [3]
I Burning [4]	U Worrying [1]
J Sore [1]	V Excruciating [5]
K Gnawing [3]	W Torturing [5]
L Pressing [4]	

#### Physical function

The KOOS and the HOOS are reliable and validated disease-specific questionnaires used to measure baseline function, pain and change over time in patients with knee and hip OA [22, 23] and consist of five joint-specific subscales: Pain; Symptoms; Activities in daily living (ADL); Function in sports and recreation; and Hip/Knee-related quality of life (QoL). Each subscale consists of questions graded 0–4 (0 equals no problems and 4 extreme problems) and the subscales are scored separately from 0 to 100 where 0 indicates worse outcome. Each subscale of the KOOS and HOOS is scored independently.

#### Health-related quality of life

The EQ-5D is a widely used and validated generic questionnaire provided by the EuroQoL group to measure HRQoL [24]. In this study, the EQ-5D VAS measure of overall health was used, with scores ranging from “best imaginable health” [100] to “worst imaginable health” [0].

### Statistics

Categorical data were described using frequencies and percentages. Continuous data were described using means with standard deviations, when normally distributed and median with range, if skewed. Patients were stratified using a cut point of 65 years, as this is the usual retirement age in Sweden (e.g. < 65 years were referred to as younger adults and those ≥65 years as older). T-tests and Mann-Whitney U tests were used, depending on data normality, to calculate differences in POM, KOOS/HOOS and EQ-5D VAS between age and sex groups. To test differences in ordinal outcomes (e.g. radiographic severity of OA and use of analgesics) and joint involved, sex or age group, a Fisher’s exact test or Chi squared test was used. A three-way ANOVA was used to test differences in KOOS/HOOS, POM-VAS and POM-Words, with age group, sex and affected joint as the independent factors. Skewed variables were log-transformed to meet the assumptions of ANOVA. All statistical tests were two-sided, with a significance level of 0.05. IBM SPSS version 22 and 23 were used for all calculations.

## Results

**Fig. 1:**
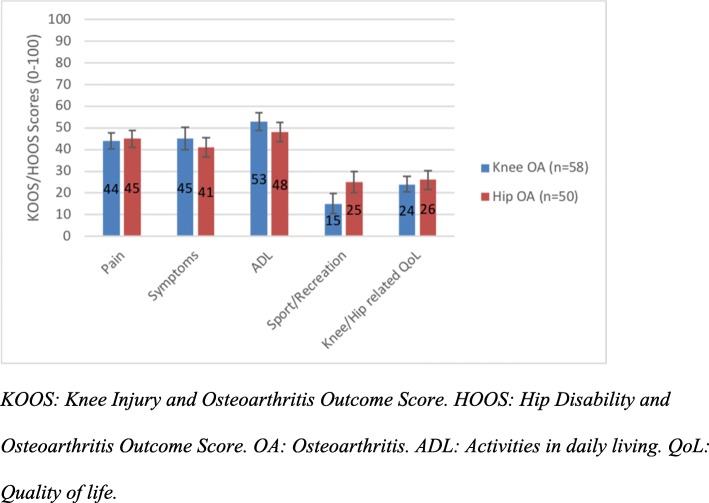
KOOS and HOOS subscales among patients with hip OA or knee OA. No difference in the dimensions of KOOS and HOOS except Sports/Recreation (*p* = 0.006) in patients with knee or hip OA. Error bars represent 95% confidence intervals

**Fig. 2:**
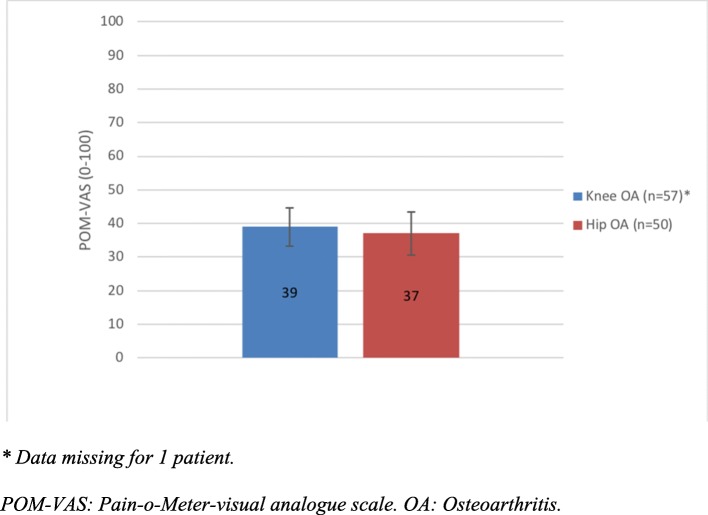
POM-VAS among patients with knee OA or hip OA. No difference in pain intensity among patients with knee or hip OA. Error bars represent 95% confidence intervals

Overall, the most common sensory words used to describe pain were grinding (B) and aching (G), and the most common affective words were irritating (M), troublesome (O) and tiring (T)*.* All patients had moderate to severe radiographic OA as noted by Kellgren & Lawrence score 3a/b or 4a/b. The mean EQ-5D VAS score was 63 (SD 21). Except for the Sports/Recreation dimension of KOOS/HOOS there were no major differences between those individuals with knee OA compared to those with hip OA in KOOS or HOOS (Fig. 1) and POM-VAS (Fig. 2).

### Differences stratified by age

Patients under 65 years of age used the words troublesome (O) and excruciating (V) more often to describe their pain than older patients. Younger patients also used significantly more affective words **(**3 (1-9) and 2 (1–7) respectively) to describe their pain, and presented with significantly greater intensity sensory scores (8 (2–20) and 6 (0–24)), and intensity affective scores (8 (3–39) and 5.5 (2–27) respectively). Younger patients scored worse for the KOOS/HOOS Symptoms subscale compared to the older (mean 38 (SD 16) and 46 (SD 19) respectively). There were no differences between age groups for the other subscales of KOOS/HOOS, EQ-5D VAS or in pain levels measured with POM-VAS. According to the modified Kellgren & Lawrence OA classification, younger patients had significantly less advanced structural OA than older patients (27 and 52% respectively with grade 4b) (Table 2).

**Table 2:** Demographic and clinical variables stratified by age group

		≥65 years of age (*n* = 64)	< 65 years of age (*n* = 44)	*p*-value	All (*n* = 108)
Joint, no (%)					
Knee OA		31 (53)	27 (47)	n.s.	58 (53.7)
Hip OA		33 (66)	17 (34)		50 (46.3)
Sex, no (%)					
Women		40 (55)	32 (45)	n.s.	72 (66.7)
Men		24 (66.6)	12 (33.3)		36 (33.3)
Age, mean (SD)		72 (5.1)	58 (5.1)		66.3 (8.5)
BMI, mean (SD)		27.6 (4.0)	29.3 (5.2)	n.s.	28.3 (4.6)
Analgesics^a^, no (%)					
Daily analgesics		24 (39.3)	24 (55.8)	0.09	48 (46.2)
Analgesics when needed		26 (42.6)	15 (34.9)		41 (39.4)
No analgesics		11 (18)	4 (9.3)		15 (14.4)
Kellgren & Lawrence modified classification of OA, no (%)					
3a		4 (6.3)	3 (6.8)	0.03	7 (6.5)
3b		12 (18.8)	16 (36.4)		28 (25.9)
4a		15 (23.4)	13 (29.5)		28 (25.9)
4b		33 (51.6)	12 (27.3)		45 (41.7)
POM VAS^b^, mean (SD)		37 (21)	40 (24)	n.s.	38 (22.6)
POM-Words					
Words, no (%)	Troublesome (O)	47 (73.4)	40 (90.9)	0.03	87 (80.6)
	Excruciating (V)	5 (7.8)	10 (22.7)	0.045	15 (13.9)
Number, median (range)	Sensory words	2 (0–7)	3 (1–6)	n.s.	2 (0–7)
	Affective words	2 (1–7)	3 (1–9)	0.02	2 (1–9)
Intensity score, median (range)	Sensory words	6 (0–24)	8 (2–20)	0.05	7 (0–24)
	Affective words	5.5 (2–27)	8 (3–39)	0.008	6 (2–39)
KOOS/HOOS, mean (SD)					
Pain		46 (14)	41 (14)	n.s.	44 (14)
Symptoms		46 (19)	38 (16)	0.03	43 (18)
ADL		52 (15)	50 (18)	n.s.	51 (16)
Sports and recreation		22 (18)	16 (18)	n.s.	20 (18)
QoL		25 (16)	24 (12)	n.s.	25 (15)
EQ-5D VAS		65 (22)	61 (19)	n.s.	63 (21)

^a^ Data missing for 4 patients ^b^ Data missing for 1 patient

*OA* Osteoarthritis, *POM* Pain-o-Meter, *VAS* Visual analogue scale, *KOOS* Knee Injury and Osteoarthritis Outcome Score, *HOOS* Hip Disability and Osteoarthritis Outcome Score, *EQ-5D VAS* EuroQol 5 Dimensions visual analogue scale, *N.s* non-significant

### Differences between women and men

With respect to pain intensity using the POM-VAS, women rated their pain higher (mean 42 (SD 24) versus 31 (SD 19) for men). Women reported they used analgesics more frequently when needed than men (45.7 and 26.5%, respectively). No differences were seen between men and women for KOOS/HOOS scores or EQ-5D VAS. There were no significant differences between men and women in how they described their pain using sensory or affective POM-words: no difference in the number of sensory or affective words used, or the sensory or affective intensity scores. Neither were any differences found in radiographic severity of OA between men and women (Table 3).

**Table 3:** Demographic and clinical variables stratified by sex

		Women (*n* = 72)	Men (*n* = 36)	*p*-value	All (*n* = 108)
Joint, no (%)					
Knee OA		38 (65)	20 (35)	n.s.	58 (53.7)
Hip OA		34 (68)	16 (32)		50 (46.3)
Age, no (%)					
≥ 65 years		40 (55)	24 (66.6)	n.s.	64 (59.3)
< 65 years		32 (45)	12 (33.3)		44 (40.7)
Age, mean (SD)		66.4 (8.9)	66 (7.8)	n.s.	66.3 (8.5)
BMI, mean (SD)		28.4 (5.1)	27.9 (3.2)	n.s.	28.3 (4.6)
Analgesics^a^, no (%)					
Daily analgesics		32 (45.7)	16 (47.1)	0.03	48 (46.2)
Analgesics when needed		32 (45.7)	9 (26.5)		41 (39.4)
No analgesics		6 (8.6)	9 (26.5)		15 (14.4)
Kellgren & Lawrence modified classification of OA, no (%)					
3a		3 (4.2)	4 (11.1)	n.s.	7 (6.5)
3b		22 (31)	6 (16.7)		28 (25.9)
4a		20 (28)	8 (22.2)		28 (25.9)
4b		27 (37.5)	18 (50)		45 (41.7)
POM VAS^b^, mean (SD)		42 (24)	31 (19)	0.011	38 (22.6)
POM-Words					
Number, median (range)	Sensory words	3 (1–7)	2 (0–5)	n.s.	2 (0–7)
	Affective words	2 (1–9)	2 (1–4)	n.s.	2 (1–9)
Intensity score, median (range)	Sensory words	7 (1–24)	6 (0–14)	n.s.	7 (0–24)
	Affective words	6.5 (2–39)	6 (2–14)	n.s.	6 (2–39)
KOOS/HOOS, mean (SD)					
Pain		42 (14)	48 (13)	0.06	44 (14)
Symptoms		41 (17)	47 (20)	n.s.	43 (18)
ADL		50 (17)	54 (14)	n.s.	51 (16)
Sports and recreation		19 (19)	22 (16)	n.s.	20 (18)
QoL		24 (14)	25 (16)	n.s.	25 (15)
EQ-5D VAS		63 (21)	65 (20)	n.s.	63 (21)

^a^ Data missing for 4 patients ^b^ Data missing for 1 patient

*OA* Osteoarthritis, *POM* Pain-o-Meter, *VAS* Visual analogue scale, *KOOS* Knee Injury and Osteoarthritis Outcome Score, *HOOS* Hip Disability and Osteoarthritis Outcome Score, *EQ-5D VAS* EuroQol 5 Dimensions Visual analogue scale, *N.s* non-significant

### Three-way ANOVA

With sex and joint involved as independent factors, no differences remained between younger and older patients with respect to KOOS/HOOS symptoms subscale, number of affective words, intensity scores for POM sensory or affective words. Patients with knee OA reported significantly higher intensity scores for sensory words used than those with hip OA (*p* = 0.015) and had worse KOOS/HOOS ADL subscale scores (*p* = 0.037) with sex and age group as independent factors. The difference between men and women in pain intensity measured with VAS remained, with joint and age as independent factors (*p* = 0.017).

## Discussion

This study aimed to investigate differences in pain expression using the POM between younger and older patients, and between men and women scheduled for a knee or hip arthroplasty, in an attempt to comprehensively describe the multifaceted concept of the pain experience. We found that patients under 65 years expressed higher pain intensity by using 10% more affective words with higher intensity values in the POM compared to the older patients. Further, younger patients scored clinically significantly worse on KOOS/HOOS symptoms [28]. However, no differences in pain as measured with POM-VAS or pain subscale of KOOS/HOOS were found between the groups. Thus, younger patients used more emotions to describe their OA pain but did not express quantitatively more pain captured by the traditionally used VAS instrument. Similar to our findings, Riley et al. [9] reported that patients with chronic pain aged 45 to 64 years expressed more intense negative emotions related to pain than those older than 65 years, despite no differences in pain intensity measured with VAS. The authors suggested that among older patients, pain may be a natural age-related phenomenon, or that differences in life circumstances may play a role in pain expression [9]. In line with this, the younger patients in this study reported more joint-related symptoms and may have demands for higher joint-related activity level and less pain due to different life circumstances compared to older and thus express more negative emotions.

In this study, younger patients scheduled for a joint arthroplasty had less radiographic severity of OA than the older patients scheduled for a joint arthroplasty. Similarly, Haynes et al. (2016) found that younger patients with knee OA awaiting surgery, had less severe radiographic OA according to the Kellgren & Lawrence score compared to older patients [6]. The association between knee-related pain and radiographic severity, in patients with OA, is weak [20] and a lower grade of radiographic OA among younger patients may be a result of age but has to be investigated further. In Sweden, pain, rather than radiographic severity, is the main indication for performing a joint replacement and all patients in this study underwent surgery despite the lower grade of radiographic OA among younger patients. A previous study showed that obese patients (> 40 kg/m^2^ in BMI) with knee or hip OA scored their pain higher [13]. We found no differences in BMI between the age groups (Table 2), that could potentially explain the difference in pain expression. Thus, we believe younger patients were more emotionally affected by their OA and expressed pain using more affective words, leading to surgery at a less radiographically advanced stage of OA.

There was a difference in pain measured with POM-VAS between sexes and in intensity scores for affective POM-words between ages. In a validation study by Gaston-Johansson et al. [21], there was a moderate to high correlation between VAS and POM-Words in patients with chronic pain due to rheumatoid arthritis. In the present study, patients were instructed to assess their joint-related pain when walking from the waiting room, which represents the current pain experience. The instructions for the KOOS/HOOS pain scale states patients are to report pain experienced within the previous week. This difference in timeframe might explain the discordance between POM-VAS, POM-Words and KOOS/HOOS and as such could be considered a limitation of this study. However, the affective component of POM-Words might capture another aspect of living with OA than merely pain, even though the patients were asked explicitly to describe the pain. The most common affective words used were irritating, troublesome and tiring and additionally for the younger patients: excruciating. We might have captured the experience of living with OA from a broader perspective (e.g. psychological) that is just as, if not more, important than only pain intensity. However, there were no difference in EQ-5D VAS, measuring the patients’ overall health, that could support this hypothesis. It would be interesting and important to further investigate the emotional aspect of pain in OA patients and whether this can influence the outcome of surgery.

Consistent with previous research [29], women in our study reported higher pain levels as measured with VAS than men. We also found that women reported more frequent use of analgesics than men, which is consistent with a previous study reporting that women across ages are prescribed more non-steroidal anti-inflammatory drugs than men [30]. This may reflect different pain coping strategies between sexes [31] or tentatively, that women are more likely to undertake preventive care and thereby, use analgesics as a way to prevent pain. Using analgesics can be considered a direct and problem-focused way of managing pain, a strategy that previously has been associated with male gender [32]. Further research on the subject is warranted. Although women scored higher on the POM-VAS, they did not describe their pain as worse by using more words with a higher intensity value for the affective component of POM-Words, indicating that they may not be more emotionally affected by their OA pain than men. With age and sex as independent factors, there remained a significant difference in intensity score for sensory POM-Words and KOOS/HOOS ADL between knee and hip OA. To our knowledge, there are no previous studies that have examined differences between patients with either knee or hip OA in intensity scores for POM sensory words. Further research is warranted on the subject.

Recently, arthroplasty registries began to focus on postoperative outcomes of TJA in terms of PROs but, as far as we know, they do not have any instrument to measure the emotional experience [1, 2]. The POM may be a useful tool in both research and clinical settings to assess pain in a more qualitative and complete way. The Osteoarthritis Research Society International (OARSI) suggested that OA should be divided into the *disease* OA representing the structural changes, and the *illness* OA representing the patient-reported symptoms, and both should be acknowledged when deciding on different treatment methods [33]. By using the POM, one could examine the *illness* by discriminating between different types of pain and provide treatment accordingly and further to identify those patients expressing more emotional distress. Hypothetically, incorporating the POM into clinical visits adds another pain dimension and may yield better communication between the patient and the healthcare provider, ultimately improving management of pain [21] and the experience of living with OA.

### Limitations and strengths

No a priori power analysis was performed as this was a new area of research and these non-significant results might be explained by the small sample size that could lead to a type II error. Low power could also explain why no differences remained between younger and older patients, with joint and sex as independent factors in a multivariable model as ANOVA. There is the potential for selection bias as we did not examine data from excluded patients, or patients who declined participation. Since we included patients with an ASA classification 1–2 suggesting overall good health, our results may not be generalizable to a larger population but may provide information on how the patients in this cohort express pain. Strengths of this study include: the use of commonly used pain outcome measures in adults with OA in the Swedish Hip and Knee Arthroplasty Registers undergoing TJA (EQ-5D VAS and KOOS/HOOS) [1, 2].

## Conclusions

Using the POM outcome measure among patients with knee and hip OA enables the evaluation of pain in a more comprehensive manner (e.g. sensory and affective components) to identify those with emotional distress. Younger patients had significantly less radiographic OA severity but had more joint symptoms than the older patients, and reported greater affective pain expression, suggesting their symptoms had greater emotional impact. Future research should focus on the emotional aspect of pain and its relation to depression/anxiety, joint-related function and postoperative results.

## Data Availability

The datasets used and analyzed during the current study are available from the corresponding author on reasonable request.

## Abbreviations

ADL: Activities of daily living
ASA: American Society of Anaesthesiologists
EQ-5D: EuroQol 5 Dimension
HOOS: Hip disability and Osteoarthritis Outcome Score
HRQoL: Health related Quality of Life
KOOS: Knee injury and Osteoarthritis Outcome Score
OA: Osteoarthritis
OARSI: Osteoarthritis Research Society International
POM: Pain-O-Meter
QoL: Quality of Life
TJA: Total Joint Arthroplasty
TKA: Total Knee Arthroplasty
VAS: Visual Analogue Scale
